# Nanoscale imaging of shale fragments with coherent X-ray diffraction

**DOI:** 10.1107/S1600576720013850

**Published:** 2020-11-30

**Authors:** Basab Chattopadhyay, Aldritt S. Madathiparambil, Fredrik K. Mürer, Pierre Cerasi, Yuriy Chushkin, Federico Zontone, Alain Gibaud, Dag W. Breiby

**Affiliations:** a PoreLab, Department of Physics, Norwegian University of Science and Technology (NTNU), Høgskoleringen 5, Trondheim, 7491, Norway; b Petroleum Department, SINTEF Industry, Trondheim, 7465, Norway; c ESRF – The European Synchrotron, 71 Avenue des Martyrs, Grenoble, 38000, France; d LUNAM, IMMM, UMR 6283 CNRS, Faculté des Sciences, Le Mans, 72085, France; e Department of Microsystems, University of South-Eastern Norway, Campus Vestfold, Borre, 3182, Norway

**Keywords:** coherent X-ray diffraction imaging, shales, 3D morphology, mineralogy, wide-angle X-ray diffraction

## Abstract

Despite the abundance of shales in the Earth’s crust and their industrial and environmental importance, their microscale physical properties are poorly understood, owing to the presence of many structurally related mineral phases and a porous network structure spanning several length scales. Here, the use of coherent X-ray diffraction imaging (CXDI) to study the internal structure of microscopic shale fragments is demonstrated. Simultaneous wide-angle X-ray diffraction (WAXD) measurement facilitated the study of the mineralogy of the shale microparticles. It was possible to identify pyrite nanocrystals as inclusions in the quartz–clay matrix and the volume of closed unconnected pores was estimated. The combined CXDI–WAXD analysis enabled the establishment of a correlation between sample morphology and crystallite shape and size. The results highlight the potential of the combined CXDI–WAXD approach as an upcoming imaging modality for 3D nanoscale studies of shales and other geological formations via serial measurements of microscopic fragments.

## Abstract 2

Combined coherent X-ray diffraction imaging and wide-angle X-ray diffraction is demonstrated to study the morphology, internal structure and mineralogy of shale fragments. Estimates of the unconnected nanoscale pore structure of shale microparticles are obtained.

## 1. Introduction   

Shales, the most abundant sedimentary rocks in the Earth’s crust, are typically made up of clay microparticles and silt-size mineral grains. Characterized by extremely low permeability in the range of tens of nanodarcys (10^−21^ m^2^), shales act as sealing caprock for oil and gas reservoirs over geological times (Neuzil, 2019 ▸; Vialle *et al.*, 2019 ▸). As such, shales are utilized in carbon capture and storage (CCS) (Bourg, 2015 ▸), groundwater remediation (Ingebritsen *et al.*, 2006 ▸) and storage of nuclear waste (Neuzil, 2013 ▸). As pointed out by Bourg (2015 ▸), these technologies which rely on shales can potentially contribute up to 70% of the global CO_2_ reduction efforts required to limit atmospheric CO_2_ in coming decades (Pacala & Socolow, 2004 ▸). The characterization of morphology and the porous structure of shales is a challenging task because of the inherent structural heterogeneity and complex mineralogy (Ma, Fauchille *et al.*, 2017 ▸). Although studied for many years, the structure–property relationship for shales remains elusive (Bourg, 2015 ▸; Dayal, 2017 ▸; Leu *et al.*, 2016 ▸; Schultz *et al.*, 1980 ▸), which poses serious engineering problems, *e.g.* for their use as a source rock or as a low-permeability barrier material.

The physical properties of shales are dependent on the grain size, mineralogy, porosity and permeability. Knowledge of the internal structure is essential to the understanding of fluid transport and storage mechanisms in shales. The presence of hydro­carbons in the subsurface is a direct consequence of the low permeability of shales over the reservoirs, trapping the hydro­carbons there despite the buoyancy forces tending to make them migrate to shallower layers. The low permeability of shales is sought after for containment of CO_2_ (Vialle *et al.*, 2019 ▸) or nuclear waste sequestration (Neuzil, 2013 ▸). However, many shales have a total porosity above 30%, comparable to permeable sandstones (Horsrud, 2001 ▸). It follows that pore connectivity and pore-size distribution are important for the macroscopic permeability properties. The transport properties must therefore be determined by considering the pore structure at the smallest scale, in the clay-rich areas where nanometre-scale pores are present (Chen *et al.*, 2013 ▸; Javadpour, 2009 ▸). Investigating shales at the shortest scale is likely to be an important step towards improving predictive models but it is expensive to obtain correctly preserved core samples from shale layers. However, the drilling process produces rock fragments called cuttings, which need to be evacuated from the well and therefore can provide valuable information on the various geological layers (Bradbury *et al.*, 2007 ▸). Recently, the interest in using cuttings from drilling campaigns for obtaining geological information has seen a revival (Carugo *et al.*, 2013 ▸; Klimova *et al.*, 2019 ▸; Stuckman *et al.*, 2019 ▸). Some index tests may be performed at the drill site, when the cuttings are ‘fresh’, meaning that desiccation has not had time to occur and thus induce cracks. These tests may include density and porosity estimations, together with more advanced tests such as continuous wave technology measurements (Nes *et al.*, 1998 ▸) to estimate the compressive strength of the geological formations.

Pores in shales can vary over length scales from nanometres to several micrometres and they are distributed heterogeneously (Leu *et al.*, 2016 ▸). Pores have been reported to be present in the vicinity of clay minerals or in contact areas between crystallites of non-clay minerals such as pyrite crystals. The distribution of pores in the shale matrix is related to mineral orientation (Leu *et al.*, 2016 ▸; Zhao *et al.*, 2019 ▸). The anisotropic pore structure in shales leads to anisotropy in flow and transport properties (Ma *et al.*, 2018 ▸). However, the complex structure of shale with respect to the size, orientation and location of the minerals and pores at different length scales makes their precise characterization a challenging task. Multiscale imaging techniques are often used to study and assess shale pore structure (Ma, Fauchille *et al.*, 2017 ▸). For example, transmission electron microscopy provides atomic-scale spatial resolution but with a limited field of view of several nanometres, while X-ray micro-computed tomography provides a large field of view (cm) but with a comparatively poor resolution (µm) (Ma *et al.*, 2018 ▸).

Coherent X-ray diffraction imaging (CXDI) is an imaging approach based on computationally reconstructing images of the object from far-field (Fraunhofer) diffraction patterns obtained with a highly coherent X-ray beam (Chapman & Nugent, 2010 ▸). The key advantage of CXDI is that 3D electron-density maps of the sample can be obtained with a high spatial resolution as good as 5 nm (Chapman & Nugent, 2010 ▸; Miao *et al.*, 2015 ▸; Sandberg *et al.*, 2013 ▸). CXDI does not require a vacuum environment, as is the case in electron microscopy, and hence reduces the challenges with the sample degradation. Over the past decade, the CXDI technique has matured, and it is currently increasingly applied for solving challenges related to environmental, biological and materials sciences. For example, structural aspects of coccolithophores (Beuvier *et al.*, 2019 ▸), polymer microcomposites (Skjønsfjell, Kleiven *et al.*, 2018 ▸; Skjønsfjell, Chushkin *et al.*, 2018 ▸), vaterite-to-calcite phase transitions in microparticles (Cherkas *et al.*, 2017 ▸) and 3D phase distribution in an olivine–iron–sulfur sample (Jiang *et al.*, 2013 ▸) have recently been reported. The recent advances in CXDI so far remain unexploited in research on shale or other geological formations. A notable exception is the structural study of sandstones where ptychographic CXDI was utilized (De Boever *et al.*, 2015 ▸).

In this article, we demonstrate the use of CXDI to study the internal structure and morphology of shale fragments. To the best of our knowledge, this study is the first application of CXDI for imaging shales. The mineralogy of the crystalline shale microparticles was studied simultaneously using wide-angle X-ray diffraction (WAXD). The combined CXDI–WAXD methodology has been demonstrated recently using single-component materials (Chushkin *et al.*, 2019 ▸). We explore the feasibility of the combined CXDI–WAXD approach as a powerful 3D imaging modality for the study of multicomponent samples. The use of CXDI, as discussed in the following, necessitates that the sample sizes are sufficiently small for successful phase retrieval. This limitation presents challenges with respect to the general representativity of the shale samples, but the reported study demonstrates the applicability of the methodology for understanding the finer structures expected to be found in shales, related to clay content and nanoscale features. We propose the combined CXDI–WAXD methodology as a promising approach to study environmentally important geological materials at the smallest scale.

## 2. Experimental   

### 2.1. Sample   

The sample rock used in this study is Pierre Shale I (PS1), extracted from an outcrop in Colorado, USA (Cerasi *et al.*, 2017 ▸). The main objective of studying PS1 was to understand its microstructure as part of our ongoing research into its potential as an analogue for typical North Sea caprock for CCS operations (Cerasi *et al.*, 2017 ▸). Fragments of PS1 with a size of 2–5 µm were obtained from a core sample by scratching the sample surface with a scalpel. The microparticles were then dispersed on the surface of an X-ray transparent Si_3_N_4_ membrane (Silson Ltd, 100 nm thickness) under ambient conditions for the CXDI measurements. Isolated particles on the membrane surface were selected using an in-line optical microscope integrated in the beamline. The selected particles (three in total) were positioned in the centre of the coherent beam and measured sequentially.

### 2.2. CXDI–WAXD measurements   

**Figure 1:**
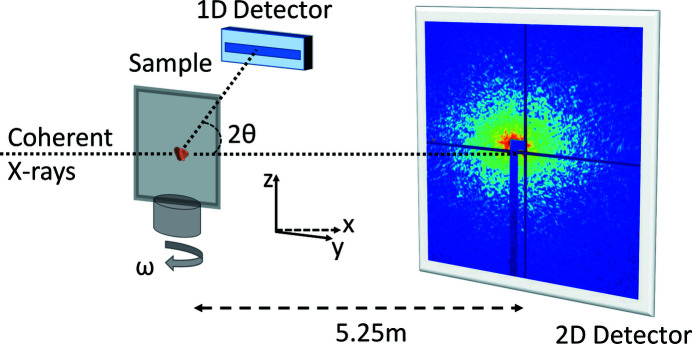
The experimental setup for simultaneous CXDI–WAXD measurements.

The CXDI–WAXD experiments were performed at the coherent scattering station of the ID10 beamline ‘ID10CS’ at the ESRF – The European Synchrotron in Grenoble, France (Chushkin *et al.*, 2014 ▸). A monochromatic 8.1 keV (λ = 1.53 Å) collimated (‘pencil shaped’) X-ray beam from a three-undulator source was used (Skjønsfjell *et al.*, 2016 ▸). The coherent primary beam from an Si(111) monochromator was selected to 10 × 10 µm (horizontal × vertical, full width at half-maximum) by rollerblade slits 50 cm upstream of the sample. Two-dimensional scattering patterns were collected using a Maxipix detector (516 × 516 pixels) (Ponchut *et al.*, 2011 ▸) with a 55 × 55 µm pixel size placed 5.25 m downstream from the sample. Correspondingly, the voxel size in real-space reconstructions is 26.1 × 26.1 × 26.1 nm. The intense direct beam was blocked by a beamstop which covered the 24 × 24 central pixels, to prevent damage to the detector. A sketch of the experimental setup is shown in Fig. 1 ▸. The scattering measurements were carried out at projection angles ω ranging from ∼−80 to 80° in steps of 0.25° with respect to the normal of the membrane surface. At each projection angle, a small-angle scattering pattern was collected on the 2D detector using an exposure time of 2.0 s. The WAXD patterns were acquired simultaneously as outlined in previous work (Chushkin *et al.*, 2019 ▸; Wallander & Wallentin, 2017 ▸) using a Mythen 1K 1D detector. The WAXD detector was placed ∼45 mm behind the sample and offset ∼40 mm from the direct beam trajectory, allowing the small-angle scattered X-rays to propagate undisturbed to the Maxipix 2D detector. The Mythen 1D detector has an active area of 50 µm × 8 mm divided into 1028 channels. The detector covered an angular range of 15–61° in 2θ. For analyses of the WAXD data, the background was subtracted and the channel number was converted to scattering angle 2θ using the known Bragg peak positions of a calibration standard Si powder. The angular resolution was 0.067 and 0.016° at 15 and 61° 2θ angles, respectively. During the tomographic ω scan, the membrane shadowed the diffracted beam near orientation angles of ω = 2θ − 90°, creating a blind range (‘missing wedge’) of ∼5° in the WAXD data. Powder diffraction data of a bulk PS1 sample were collected at the ID15A beamline at ESRF (wavelength λ = 0.248 Å).

### 2.3. CXDI reconstruction   

In CXDI an isolated microscopic object is illuminated by a plane wave with a transverse coherence length larger than the object; this results in a speckled diffraction pattern which is recorded by the detector. The recorded far-field diffraction intensity (see Fig. S1 in the supporting information for an example of the diffraction pattern for the studied samples) is proportional to the square of the modulus of the Fourier transform (FT) of the electron density of the scattering object ρ(**r**), *i.e. I*(**q**) ∝ |*F*(**q**)|^2^. Here, *F*(**q**) = |*F*(**q**)|exp[−*iφ*(**q**)] = FT[ρ(**r**)], and **q** is the scattering vector. Knowledge of the phase, φ(**q**), at the detector plane is necessary for retrieving the real-space image, but the phase information is lost in diffraction experiments because only intensities can be measured. However, for coherent radiation, the phase of the scattered field can be reconstructed using iterative numerical algorithms relying on appropriate constraints (Miao *et al.*, 2015 ▸, 1999 ▸). In reciprocal space, the calculated scattering amplitude is constrained to equal the square root of the measured intensity. In real space, a support defines regions containing nonzero electron density, outside of which the density is forced to be zero. The initial loose support is refined using the shrink-wrap algorithm (Marchesini *et al.*, 2003 ▸). This computational phase-retrieval process can be solved by several known algorithms; in this article, the hybrid input–output with error reduction algorithm was used (Chushkin *et al.*, 2014 ▸; Fienup, 1982 ▸; Miao *et al.*, 2015 ▸). The underlying fundamental principle that allows the phase-retrieval algorithm to converge is that the phase of a 2D or 3D object is uniquely coded in coherent diffraction patterns that are sampled at least twice finer than the Nyquist frequency, known as the oversampling criterion (Miao *et al.*, 1999 ▸, 2015 ▸; Sayre, 1991 ▸). In practice, it requires that the detector pixel size *p* is about three times smaller than the size of speckles in the diffraction pattern. Consequently, to fulfil the oversampling condition, the sample size, *s*, is given by 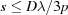, where *D* is the sample-to-detector distance and λ is the X-ray photon wavelength. Hence, in our experimental setup (*D* = 5.25 m, λ = 1.53 Å and *p* = 55 µm), samples smaller than ∼5 µm fulfil the condition for oversampling by giving speckles in the diffraction pattern that extend over several detector pixels (representative diffraction patterns of the three samples are shown in Fig. S1).

**Figure 2:**
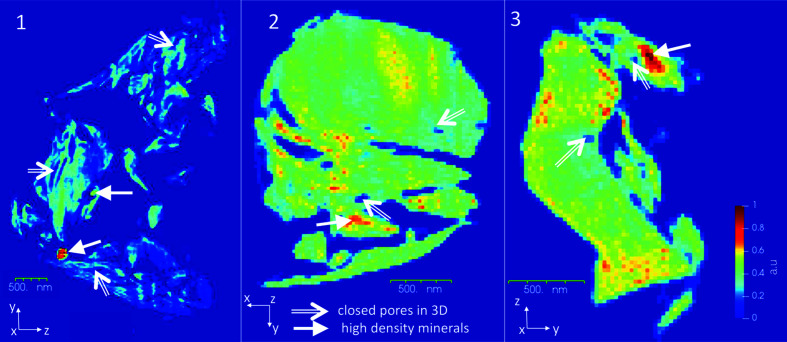
Electron-density cross sections through samples 1–3, highlighting the high-density regions and closed pores. Numerous larger pores connected to the particle exterior are easily seen.

Convergence of the iterative algorithm was reached after ∼1000 iterations, and 20 3D reconstructions were averaged to reduce noise and smooth random high-frequency variations. The spatial resolution of the final images was estimated using the phase-retrieval transfer function (PRTF) (Chapman *et al.*, 2006 ▸) shown in Fig. S2. A PRTF value of 0.5 was used as a criterion to estimate the spatial resolution. Accordingly, we found 29.6, 36.3 and 55.2 nm resolutions for samples 1, 2 and 3, respectively. The variation in the resolution is a direct consequence of the variation in the size of the samples. The smaller the sample the weaker the scattered signal (Fig. S1) and hence the poorer the resolution. In our study, sample 1 was the largest and sample 3 was the smallest. The size of the sample also had an impact on the low-frequency density variations (see Fig. 2 ▸). In the reconstructed CXDI images, artefacts in the form of over- or underestimation of the electron densities are present. These artefacts can at least partially be attributed to the fact that the scattered intensities near the direct beam, corresponding to the lowest spatial frequencies, were not measured in our CXDI experiment (Skjønsfjell, Kleiven *et al.*, 2018 ▸; Thibault *et al.*, 2006 ▸). We estimated that 10, 4 and 1 central speckles were missing for samples 1, 2 and 3, respectively. The reconstructed images were processed by subtracting background noise and setting all negative density values to zero. Image processing and analyses were carried out using *Tomviz* (Hanwell *et al.*, 2019 ▸) and *Avizo* (2018 ▸). Representative scanning electron microscopy (SEM) images and corresponding energy-dispersive X-ray spectroscopy (EDS) spectra are shown in Figs. S3 and S4. SEM shows the surface morphology and does not allow characterization of the internal porosity and crystalline phase determination. Hence correlation between CXDI and the SEM images is difficult and comparison with SEM images does not provide additional information. CXDI is a reliable imaging modality and has been demonstrated to be accurate in numerous studies during the past two decades (Chapman & Nugent, 2010 ▸; Chushkin *et al.*, 2019 ▸).

## 3. Results and discussion   

CXDI allows studying the surface morphology of the samples in addition to their internal porous structure. The 2D electron-density maps of samples 1–3 depicted in Fig. 2 ▸ show the high-density regions and closed pores (see the white arrows). The most distinctive feature in sample 1 is the presence of two spheroidal inclusions, both with a diameter of ∼200 nm. The relative electron density corresponding to these inclusions is about twice that of the rest of the sample, consistent with the presence of pyrite (FeS_2_) as the inclusion mineral. Similarly, in samples 2 and 3, one can identify high-density regions and pore spaces. Spheroidal pyrite crystals, as in sample 1, were not observed in samples 2 and 3 but the presence of pyrite can be ascertained from the WAXD data discussed later. From Fig. 2 ▸ one can also conclude the presence of lower-density minerals, notably clay minerals and/or organic content in sample 1. Conversely, in samples 2 and 3, the electron-density distribution is rather uniform throughout the entire sample, consistent with the reported presence of quartz, illite, albite or orthoclase. (See Movies S1–S3 in the supporting information.)

**Figure 3:**
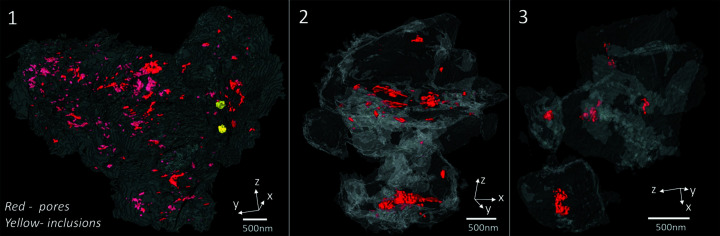
3D iso-surface renderings of samples 1–3 with the high-density inclusions (only visible in sample 1) and 3D closed pores shown.

Three-dimensional isosurface renderings (see also Movies S1–S3) highlighting the pore structures of all the samples are depicted in Fig. 3 ▸. The closed pore volumes are estimated to be 0.31 (5), 0.43 (4) and 0.39 (7) vol.% in samples 1, 2 and 3, respectively. By ‘closed pores’ we refer to porous structures enclosed within the 3D structure and thus not connected to the external sample surface. The spatial distribution of the closed pores is non-uniform, as the pores are observed to be localized to certain regions of the sample and they do not display a connected pore network (Fig. 3 ▸). The characteristic diameter of the observed closed pores, as estimated from the 3D CXDI data sets, varied from 50 to 200 nm.

In shales the presence of closed pores has been observed in the vicinity of high-density minerals like pyrite, as seen in sample 2, or within the clay matrix, as in sample 1 (*cf*. Fig. 2 ▸) (Ma, Fauchille *et al.*, 2017 ▸). The pore structure observed here can be classified as the inter-mineral pores that appear at the grain boundaries between mineral phases and/or intra-mineral pores, which are present in agglomerates of minerals such as pyrite and dolomite (Ma *et al.*, 2016 ▸, 2018 ▸). However, it is difficult to classify the pore structure conclusively owing to challenges in preparing the small and brittle samples, the inherent inability of CXDI imaging to distinguish between different mineral phases of similar density, and insufficient resolution of the data sets.

In order to estimate the mineralogy of the PS1 sample, a Rietveld refinement (Rietveld, 1969 ▸) of the powder diffraction pattern from bulk samples was carried out using the *GSAS-II* software package (Toby & Von Dreele, 2013 ▸). The corresponding refinement plot is given in Fig. S5. As seen from Fig. S5, several Bragg peaks overlap, giving a considerable uncertainty in the concentration estimate for the low-concentration minerals. The quantitative phase analyses revealed that the most dominant mineral present in the sample is quartz, along with clays and feldspar, which is consistent with previous reports (Kuila & Prasad, 2013 ▸; Schultz *et al.*, 1980 ▸). The clay minerals can be identified as illite, kaolinite, clinochlore and montmorillonite, while the feldspar group is represented by albite and orthoclase. Minor amounts of pyrite and dolomite were also identified.

**Figure 4:**
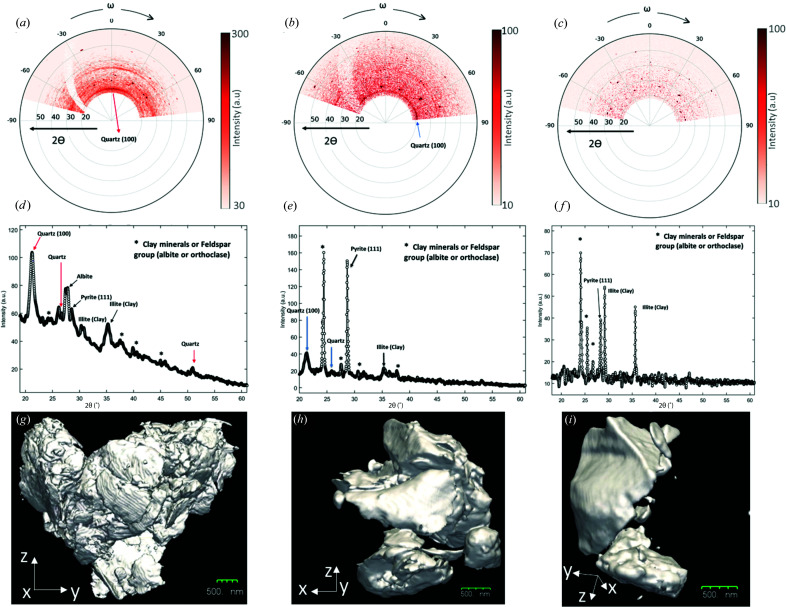
(*a*)–(*c*) Polar maps of diffraction data with the diffracted intensity plotted as a function of the 2θ angle (radius) and the sample rotation angle ω for samples 1, 2 and 3, respectively. (*d*)–(*f*) Corresponding 1D diffraction data obtained by integrating over ω. (*g*)–(*i*) 3D iso-surface renderings of the CXDI reconstruction for samples 1, 2 and 3, respectively.

The simultaneously recorded WAXD measurements provide valuable insights into the minerals present in the microparticles studied here. Figs. 4 ▸(*a*)–4(*c*) show polar diffraction maps with the diffracted intensity plotted as a function of the 2θ angle (radius) and the sample rotation angle ω (azimuth). Figs. 4 ▸(*d*)–4(*f*) depict the corresponding 1D diffraction profiles obtained by averaging over ω. Indexing of the Bragg peaks was carried out using these 1D diffraction profiles. The presence of the quartz 100 diffraction ring is apparent in samples 1 and 2, as seen in Figs. 4 ▸(*a*) and 4(*b*). In sample 1, a coincidentally in-plane wide orientation (with respect to the Si_3_N_4_ membrane) of the (100) plane of quartz is observed, whereas in sample 2 the corresponding orientation is uniform but grainier. Other peaks in the diffraction patterns can be attributed to the clay or feldspar minerals present in the shale structure. The 111 peak of pyrite can be indexed in all three diffractograms, confirming the presence of pyrite in PS1. Although present in trace amounts, the pyrite contained in the samples can be distinguished by its characteristic spheroidal geometry (Er *et al.*, 2016 ▸; Scott *et al.*, 2009 ▸), as seen in Figs. 2 ▸ and 3 ▸. The presence of pyrite in the form of clusters of small crystallites has been reported in other shale specimens using electron microscopy (Ma, Fauchille *et al.*, 2017 ▸; Rodriguez *et al.*, 2014 ▸). Figs. 4 ▸(*g*)–4(*i*) show the corresponding 3D iso-surface renderings of the three samples (see Movies S1–S3).

Distinct relations between the sample morphology and the 1D diffraction patterns were observed. Sample 1 is composed of numerous small crystallites with sizes less than 1 µm and this is manifested in the 1D diffraction pattern as broad peaks. The broad 100 quartz peak implies the presence of small quartz crystallites of size ∼11 nm (as estimated using the Scherrer formula, Fig. S7) in both samples 1 and 2. The absence of the characteristic quartz 100 diffraction peak in sample 3 can be attributed to either (i) the absence of quartz in the sample or (ii) the presence of only one or a few quartz crystallites oriented such that no diffraction peaks were recorded. In samples 2 and 3, the Bragg peaks corresponding to other mineral phases are sharper owing to the presence of larger crystallites. In sample 3, large crystallites with sharp facets are evident in the CXDI reconstruction [*cf*. Fig. 4 ▸(*i*)].

The complementary information obtained from the 3D CXDI images and the corresponding WAXD data could be utilized to study phase information in multiphase objects, and crystal shape and orientation information. With the current experimental setup, only a limited quantitative analysis of the WAXD data could be carried out as the 1D detector covers only a small solid angle. Moreover, the step size of 0.25° in the scanned projection angle ω is large compared with the intrinsic width of the crystallite Bragg peaks. Future experimental setups will be improved to facilitate correlation between the 3D CXDI images and shape/orientation information of the constituent crystallites.

The size of the sample in this combined CXDI–WAXD approach is dictated by the oversampling condition that must be satisfied for the phase-retrieval algorithm to converge. Larger samples will give smaller speckles, covering fewer detector pixels (see Fig. S1). Oversampling can be improved by increasing the sample-to-detector distance, but the spatial resolution of the reconstruction will then degrade owing to the reduced numerical aperture (finite detector size). The achievable resolution is thus dependent on the sample-to-detector distance. The recently finished upgrade of the ESRF (extremely brilliant source, EBS) (ESRFnews, 2017 ▸), together with new large-array pixelated 2D detectors, is expected to improve these aspects by providing a higher resolution in three dimensions (<5 nm), a larger field of view (10–15 µm) and the possibility of time-resolved studies.

It is instructive to compare CXDI with X-ray phase nano-computed tomography (nanoCT), which also relies on a highly coherent X-ray beam and offers higher sensitivity than conventional X-ray computed tomography (Cloetens *et al.*, 1999 ▸; Mokso *et al.*, 2007 ▸). While CXDI as described is based on reconstructing a real-space image from Fraunhofer diffraction patterns, phase nanoCT relies on propagation-based phase contrast in the Fresnel regime (Cloetens *et al.*, 1999 ▸). For the latter technique, one or more raw images at different focus-to-sample distances are analysed to numerically retrieve one optimal image through the Paganin method (Paganin *et al.*, 2002 ▸). NanoCT enables imaging of larger samples of ∼100 µm, albeit with a lower resolution of ∼50–100 nm, as dictated by the depth-of-field limitation (Tsai *et al.*, 2016 ▸), and arguably with quantitatively less accurate phase contrast as Paganin’s approach is based on homogeneous single-component samples (Häggmark *et al.*, 2017 ▸; Hehn *et al.*, 2018 ▸). For 3D CXDI, the depth of field is not a concern, because the resolution is governed by the maximum scattering angle. For samples with high angles of scattering, the size of the detector limits the maximum scattering angle and hence the resolution. Moreover, in CXDI sample vibration does not affect the resolution as the probe is a plane wave. In contrast to nanoCT, CXDI is more suited for high-resolution imaging of small (<6 µm) samples where the sample size is limited by the available sample-to-detector distance.

Another concern is to what extent the samples are representative of the larger core sample or, ultimately, of the entire geological formation. This question includes the arbitrariness in the sample preparation and selection. Clearly, sample preparation by focused-ion-beam milling (Trtik *et al.*, 2013 ▸) should be attempted to achieve well defined sample geometries at selected regions of interest. The upcoming larger field of view (ESRFNews, 2017 ▸) will also reduce the current ambiguities relating to whether the extracted micrometre-scale samples are in fact grains that are comparatively hard with respect to the surrounding matrix, and also whether the pores observed here as ‘open’ are in fact part of larger ‘closed’ pores that represent a weak zone through the material.

With the mentioned upcoming experimental improvements relating to the EBS source, we envision a measurement scheme where a large number of rock fragments (say, 10^1^–10^4^) are sequentially imaged by CXDI in a fully automated fashion along with precise corresponding WAXD measurements. With this approach, the different advantages of CXDI and X-ray diffraction microscopy (Mürer *et al.*, 2018 ▸; Poulsen, 2012 ▸) will work together, giving high-resolution imaging with mineral specificity besides providing information about crystal orientation and interlayer spacing. With these considerations in mind, we advocate the combined CXDI–WAXD approach as a promising imaging modality for the nanoscale study of shales and other complex geological structures.

## 4. Conclusions   

In this article, we have demonstrated the combined use of CXDI and WAXD to study the morphology, internal structure and mineralogy of Pierre Shale I. It was possible to directly localize pyrite nanocrystals as inclusions in the quartz–clay matrix. The volume percentage of closed pores was estimated to be in the range of 0.3–0.4%, which corroborates the reported porosity data for shales (Ma, Taylor *et al.*, 2017 ▸). The combined CXDI–WAXD analysis enabled us to establish a correlation between sample morphology and crystallite shape and size. The methodology proposed here opens possibilities for quantitative geological and petrophysical analyses on small samples such as drill cuttings, removing the need for large cores which are seldom taken from caprock shales.

The experimental and the reconstructed data for samples 1–3 are available freely through the UNINETT Sigma2 repository – https://doi.org/10.11582/2019.00044.

## Supplementary Material

Supporting information. DOI: 10.1107/S1600576720013850/gj5244sup1.pdf

Movie S1. DOI: 10.1107/S1600576720013850/gj5244sup2.mp4

Movie S2. DOI: 10.1107/S1600576720013850/gj5244sup3.mp4

Movie S3. DOI: 10.1107/S1600576720013850/gj5244sup4.mp4

The experimental and the reconstructed data for samples 1-3 are available freely through the UNINETT Sigma2 repository: https://doi.org/10.11582/2019.00044
