# Supplementary material for: Nanoscale imaging of shale fragments with coherent X-ray diffraction
Source: J Appl Crystallogr. 2020 Nov 30;53(Pt 6):1562–9. doi: 10.1107/S1600576720013850 (PMC7710485; doi:10.1107/S1600576720013850)
Supplement: Supplementary file 1 [file j-53-01562-sup1.pdf]

JOURNAL OF
APPLIED
CRYSTALLOGRAPHY

Volume 53 (2020)

Supporting information for article:

Nanoscale imaging of shale fragments with coherent X-ray diffraction

Basab Chattopadhyay, Aldritt S. Madathiparambil, Fredrik K. Mürer, Pierre Cerasi, Yuriy Chushkin, Federico Zontone, Alain Gibaud and Dag W. Breiby

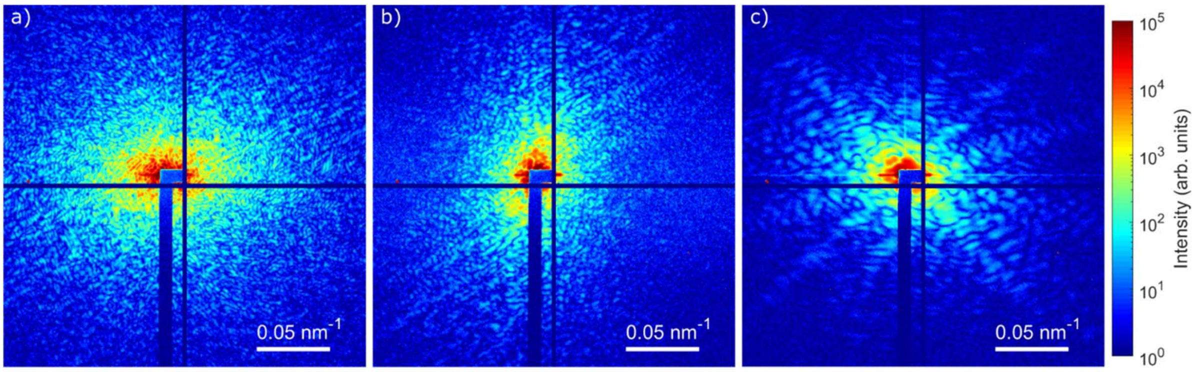

Figure S1 Representative far-field diffraction patterns for : (a) sample 1 corresponding to sample size of ~5µm, (b) sample 2 with size of ~3µm and (c) sample 3 with a size of ~2µm. Sample 1 has the strongest scattering signal and smallest speckle size while in sample 3 the scattered signal is weak and the speckle sizes are biggest.

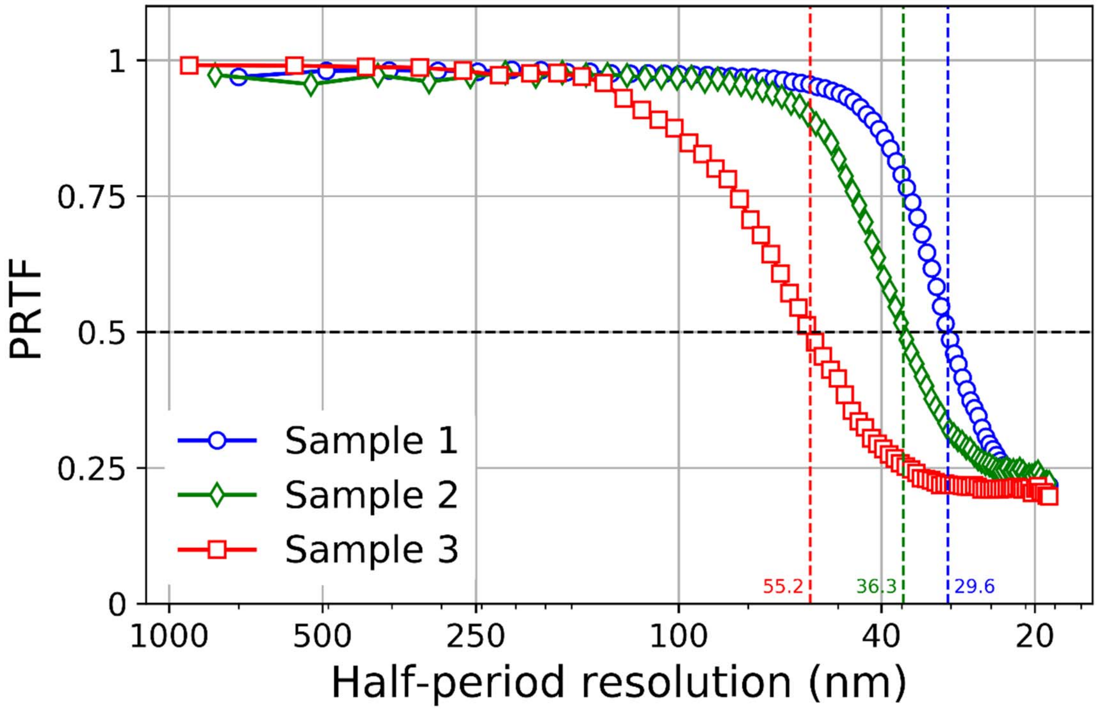

Figure S2 PRTF evaluated from the iterative phase retrieval process for samples 1-3. The positions used to estimate the spatial resolution are shown with dotted lines.

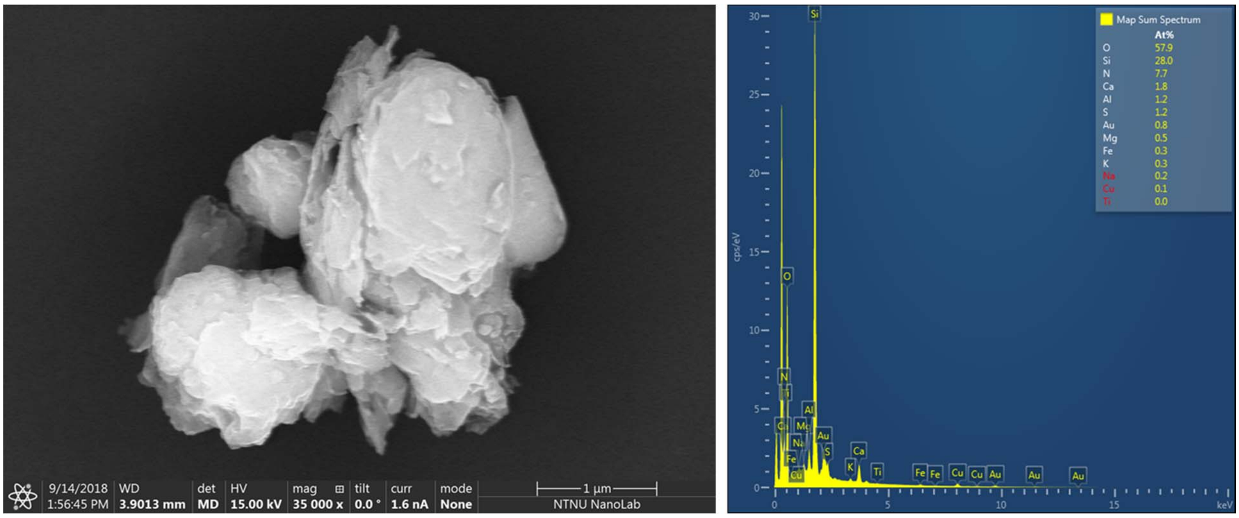

Figure S3 SEM image and the corresponding EDS spectrum for a ~4µm shale fragment.

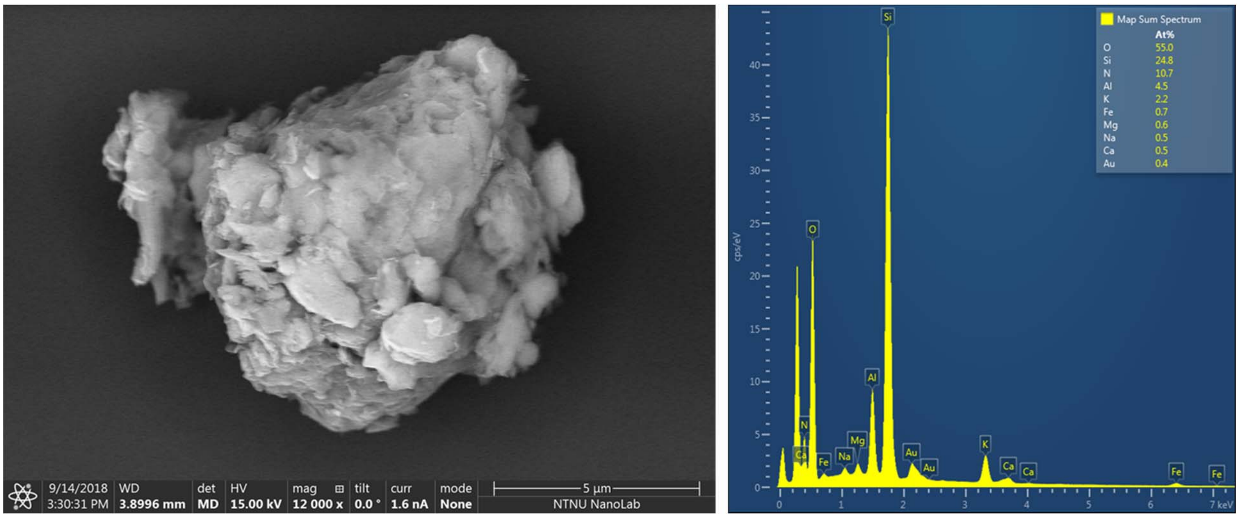

Figure S4 SEM image and the corresponding EDS spectrum for a ~10µm shale fragment.

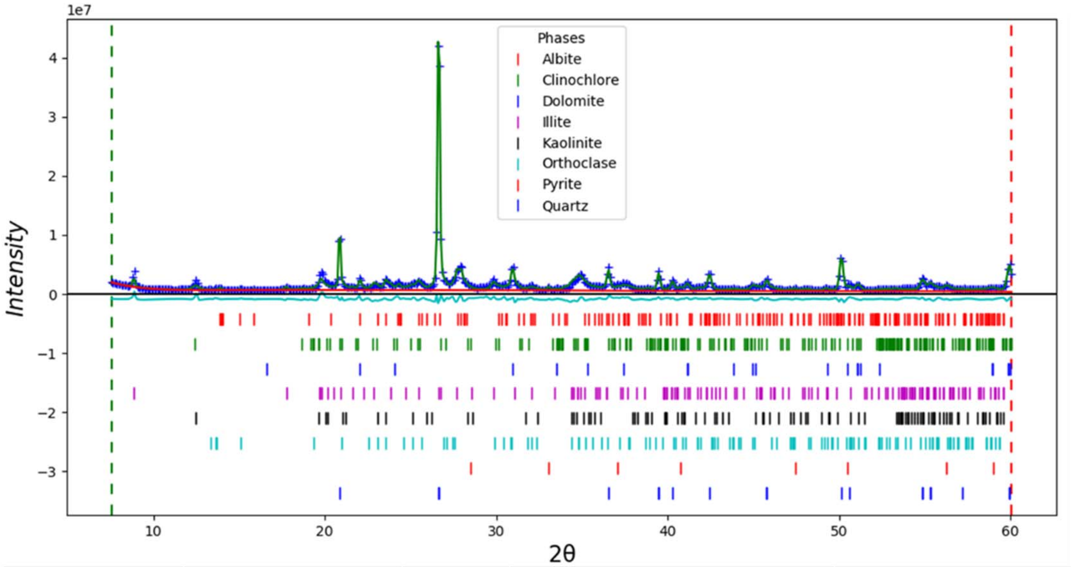

Mineral class	Mineral	Wt %	Chemical Formula	Mass Density (g/cm^3)
	**Quartz**	**34(1)**	SiO_2	2.65
Clay	**Illite**	**30(1)**	$K Al_4Si_2O_{12}$	2.78
	**Kaolinite**	**5(1)**	$Al_2Si_2O_9H_4$	2.59
	**Montmorillonite**	**Traces**	$Al_2\ Si_4\ O_{12}\ Ca_{.5}$	1.80
	**Clinochlore**	**6 (1)**	$Mg_{4.5}\ Fe_{0.5}Al_{1.84}\ Si_{3.16}\ O_{18}H_8$	2.71
Feldspar	**Albite**	**12(1)**	$NaAlSi_3O_8$	2.62
	**Orthoclase**	**7(1)**	$KAlSi_3O_8$	2.56
Carbonate	**Dolomite**	**5(1)**	$CaMgC_2O_6$	2.84
	**Pyrite**	**1(1)**	FeS_2	5.01

Figure S5 Rietveld refinement plot (top) and the corresponding phase analyses for a bulk sample of PS1. The powder diffraction data was collected at the ESRF ID15A beamline.

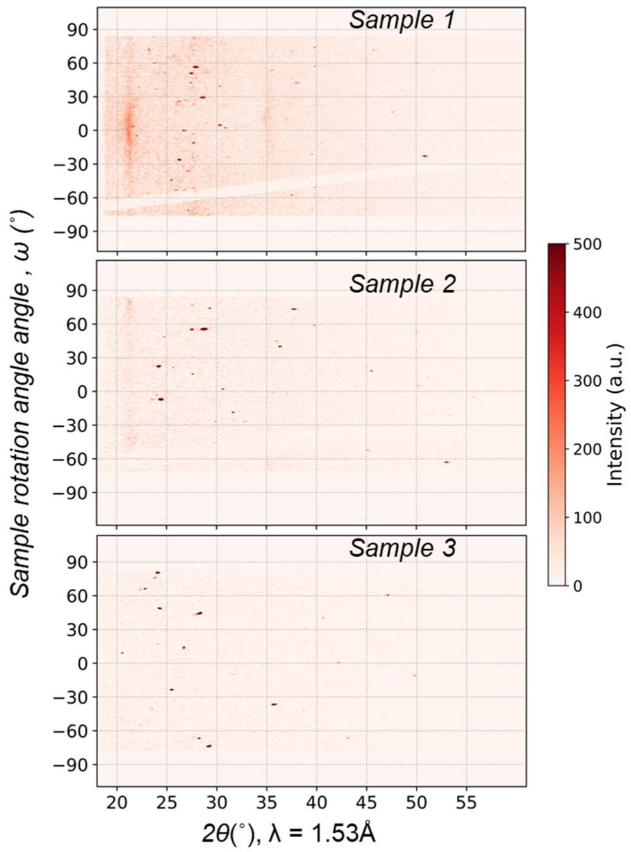

Figure S6 WAXD datasets for samples 1-3 with the sample rotation angle ω (˚) plotted as function of 2θ (˚).

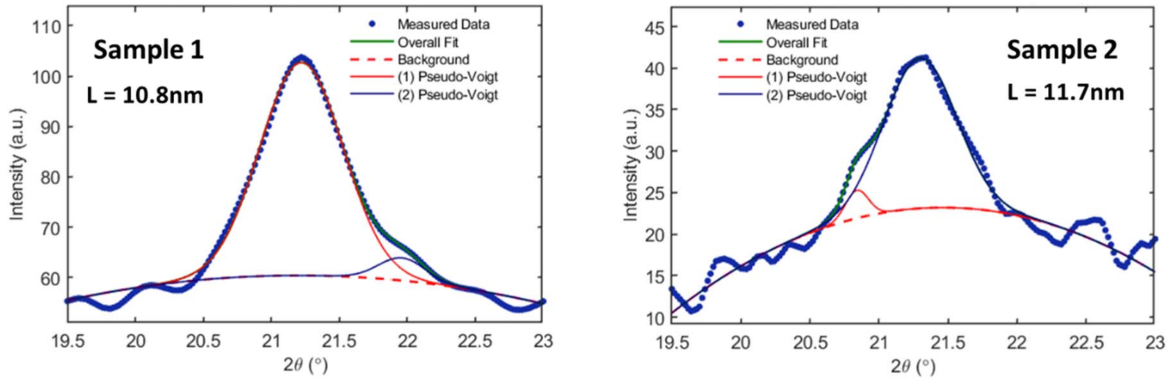

Figure S7 Crystallite size (L) corresponding to the quartz *100* peak at $2\theta \approx 21.19˚$. Fitting of the peak was done in LIPRAS (Web Page: https://github.com/SneakySnail/LIPRAS) using a pseudo-Voigt peak profile. Peak position and FWHM obtained from the fitting were used in the Scherrer formula to estimate the crystal size.

Movie S1-S3 3D isosurface view for samples 1-3 respectively with the high-density regions (pyrite minerals) shown in yellow within the transparent quartz-clay matrix.
